# Cytoglobin is upregulated by tumour hypoxia and silenced by promoter hypermethylation in head and neck cancer

**DOI:** 10.1038/sj.bjc.6605121

**Published:** 2009-06-30

**Authors:** R J Shaw, M M Omar, S Rokadiya, F A Kogera, D Lowe, G L Hall, J A Woolgar, J Homer, T Liloglou, J K Field, J M Risk

**Affiliations:** 1 Molecular Genetics and Oncology Group, School of Dental Sciences, University of Liverpool, Liverpool L69 3GN, UK; 2 Department of Surgery and Oncology, School of Cancer Studies, University of Liverpool, 5th Floor UCD, Duncan Building, Daulby Street, Liverpool L69 3GA, UK; 3 Regional Maxillofacial Unit, University Hospital Aintree, Longmoor Lane, Liverpool L9 7AL, UK; 4 Department of Oral Pathology, University of Liverpool Dental Hospital, Pembroke Place, Liverpool L3 5PS, UK; 5 University Department if Otolaryngology and Head and Neck Surgery, University of Manchester, Manchester Royal Infirmary, Oxford Road, Manchester M13 9DL, UK; 6 University of Liverpool Cancer Research Centre, Roy Castle Lung Cancer Research Programme, 200 London Rd, Liverpool L3 9TA, UK

**Keywords:** *CYGB*, head and neck squamous cell carcinoma, hypoxia, *HIF1-á*, methylation

## Abstract

**Background:**

Cytoglobin (Cygb) was first described in 2002 as an intracellular globin of unknown function. We have previously shown the downregulation of cytoglobin as a key event in a familial cancer syndrome of the upper aerodigestive tract.

**Methods:**

Cytoglobin expression and promoter methylation were investigated in sporadic head and neck squamous cell carcinoma (HNSCC) using a cross-section of clinical samples. Additionally, the putative mechanisms of Cygb expression in cancer were explored by subjecting HNSCC cell lines to hypoxic culture conditions and 5-aza-2-deoxycitidine treatment.

**Results:**

In clinically derived HNSCC samples, CYGB mRNA expression showed a striking correlation with tumour hypoxia (measured by HIF1A mRNA expression *P*=0.013) and consistent associations with histopathological measures of tumour aggression. CYGB expression also showed a marked negative correlation with promoter methylation (*P*=0.018). In the HNSCC cell lines cultured under hypoxic conditions, a trend of increasing expression of both CYGB and HIF1A with progressive hypoxia was observed. Treatment with 5-aza-2-deoxycitidine dramatically increased CYGB expression in those cell lines with greater baseline promoter methylation.

**Conclusion:**

We conclude that the *CYGB* gene is regulated by both promoter methylation and tumour hypoxia in HNSCC and that increased expression of this gene correlates with clincopathological measures of a tumour's biological aggression.

Cytoglobin (*CYGB*) is a candidate tumour-suppressor gene on chromosome 17q25 and is the only gene completely contained within the 42.5-kb Tylosis with Oesophageal Cancer (TOC) minimal region (Langan *et al*, 2004). *CYGB* is a recently described, intracellular globin (Burmester *et al*, 2002, 2004) whose function was, until recently, unknown (Trent III and Hargrove, 2002). Phylogenetic principles suggest that it has a common ancestral origin with vertebrate myoglobins (Hankeln *et al*, 2005). TOC is a rare, autosomal dominantly inherited skin condition associated with an increased risk of squamous cell oesophageal cancer and oral lesions (Tyldesley, 1974; Ellis *et al*, 1994). The 17q25 TOC minimal region is subject to frequent allelic imbalance in sporadic oesophageal cancer and *CYGB* is dramatically downregulated in affected (non-malignant) tylotic oesophageal tissue compared with normal oesophagus (McRonald *et al*, 2006). The mechanism of downregulation of *CYGB* in TOC is unclear, as no mutations have been identified in affected tissue (Shahabi *et al*, 2004), although there is evidence that *CYGB* promoter methylation may occur in sporadic oesophageal cancer (McRonald *et al*, 2006) and possibly also in ovarian cancer (Presneau *et al*, 2005).

Our recent study of *CYGB* promoter methylation in head and neck squamous cell carcinoma (HNSCC) using quantitative pyrosequencing methylation analysis (PMA) shows that methylation is both common (65% of cases) and significantly tumour-specific (*P*=0.02) in cancers at this site (Shaw *et al*, 2006). Furthermore, *CYGB* expression has recently been shown by us to be significantly downregulated in 54% sporadic non-small cell lung cancer (NSCLC) in comparison with surrounding ‘normal’ tissues (Xinarianos *et al*, 2006). The potential utility of CYGB promoter methylation as a molecular biomarker in NSCLC (Shivapurkar *et al*, 2008) and HNSCC (Shaw *et al*, 2007) has also been explored. Tumour suppressor activity of CYGB has recently been shown in NSCLC and breast cancer cell lines (Shivapurkar *et al*, 2008). Exactly what function the Cygb protein may have in aerodigestive tract squamous malignancy has not yet been elucidated.

The exact physiological role of Cygb is currently speculative, although intracellular oxygen storage, peroxidase function, oxygen sensing or binding and detoxification of nitric oxide have all been suggested (Burmester *et al*, 2002, 2004; Trent III and Hargrove, 2002; Hankeln *et al*, 2005; Hodges *et al*, 2008). It has been recently shown that upregulation of *CYGB* in rat liver cells has a protective effect against damage-induced fibrosis (Xu *et al*, 2006) and this has led to the hypothesis that *CYGB* has a homoeostatic effect, inhibiting free radical-induced fibroblast activation and consequent fibrosis. Hyperplasia of tissues resulting from downregulation of *CYGB* might conceivably constitute the first step in malignant transformation, although this suggested mechanism is speculative.

The role of Cygb in the cellular response to hypoxia may also be of significance in established malignancy. Initial speculation on the function of Cygb in hypoxia was generated not only from its structural similarity to oxygen transport molecules, but also from its localisation in areas of poor vascularity such as the brain and retina (Ostojic *et al*, 2006; Li *et al*, 2007). Subsequent studies on hippocampal cell lines confirmed that hypoxia upregulates *CYGB* mRNA expression in a ‘dose-dependent’ manner (Fordel *et al*, 2004a, 2004b). Further work on *HIF1A* (+/1) knockout mice confirmed that this effect is mediated by the *HIF1A* pathway and also that the *CYGB* gene contains hypoxia responsive elements and mRNA stabilisation sites (Fordel *et al*, 2004a). Hypoxia is a known endogenous marker of poor prognosis in HNSCC. In particular it seems to be related to resistance to radiotherapy (Koukourakis *et al*, 2006) and chemotherapy. The cellular response to hypoxia seems to be mediated by *HIF1A* in malignancy (Maxwell, 2005) and has been extensively studied in HNSCC (Brennan *et al*, 2005; Quintero *et al*, 2006).

In the current study, we measured *CYGB* gene expression and promoter methylation in a series of head and neck cancer tumours and determined associations with histopathology, clinical characteristics and established markers of tumour hypoxia such as *HIF1A* expression and tumour thickness. We also investigated the *CYGB* and *HIF1A* response of human oral tumour cell lines to hypoxic conditions.

## Materials and methods

### Patient cohort and tissue procurement

Tumour samples (5 mm^3^) were taken at the time of operation from 37 patients with biopsy-proven oral or oropharyngeal squamous cell carcinoma and snap frozen in liquid N_2_. Surgical margin samples were collected from seven of these patients at the same time for use as ‘normal’ (i.e. histologically tumour-free) samples. Selection criteria included the intention to treat by primary surgery and the absence of previous similar malignancy or treatment. Detailed tumour and patient characteristics were documented, including clinical and pathological TNM staging.

The human tissue that formed the basis for this research was collected under ethical approvals (Central Liverpool) LREC 07/Q1505/15 (7 March 2007) and (South Sefton) EC.47.01 (1 July 2002 amended on 1 March 2006) and patients gave informed consent in all cases.

### Sample preparation

DNA was extracted from 2-mm^3^ frozen tissue using DNeasy (Qiagen Ltd, Crawley, West Sussex, UK). Bisulphite treatment of 2 *μ*g of each DNA sample was undertaken using the EZ DNA Methylation Kit (Zymo Research Corporation, Orange, CA, USA) before methylation analysis.

RNA was prepared from additional 2-mm^3^ frozen tissue specimens using RNeasy (Qiagen Ltd). Adequate RNA quality and concentration was confirmed using RNA Labchip with a 2100 Bioanalyser (Agilent Technologies Inc, Santa Clara, CA, USA). cDNA was prepared from 1–2 *μ*g of RNA using RETROscript two-step reverse transcriptase PCR with random decamers (Ambion, Applied Biosystems, Warrington, UK). The quality of resultant cDNA and absence of contamination was confirmed by PCR using *β*-actin primers.

Positive and negative controls were prepared from human cell lines known to be 100% methylated (CRL5935) and 100% unmethylated (HNBE) at the *CYGB* promoter.

### Quantitative pyrosequencing methylation analysis

Pyrosequencing methylation analysis was carried out as previously described (Shaw *et al*, 2006). Briefly, hot start PCR was carried out using 100 ng of bisulphite-treated DNA template. Pyrosequencing was carried out using the PSQ96MA System (Biotage, Qiagen Ltd) according to manufacturer's protocol, including single strand-binding protein (PyroGold reagents, Qiagen Ltd). An average methylation index (MtI) was calculated from the mean of the four CpG sites evaluated as previously described.

### Real-time mRNA expression assays

Real-time PCR reactions were carried out using the relative quantification software supplied with the Applied Biosystems 7500. The target genes were *CYGB* and *HIF1A*, using FAM/MGB-labelled Taqman probes (Hs00370478_m1 and Hs00153153_m1, respectively; Applied Biosystems). The endogenous control ‘housekeeping gene’ was TATA-box-binding protein (Hs99999910_m1), as this was less likely to be affected by hypoxia than other possible control genes (Jogi *et al*, 2004; Fink *et al*, 2008). Each reaction was carried out in duplicate and the mean result was used. Relative gene expression was calculated using the 2^−ΔΔC^_T_ method (Livak and Schmittgen, 2001). The values for each tumour-derived sample were subsequently normalised relative to the mean of the seven histologically tumour-free epithelial samples taken from the limits of surgical resections.

### HIF1A immunohistochemistry

FFPE tumour blocks were available in 32 of the clinical cases and presence of carcinoma was confirmed. Immunohistochemical detection of HIF1A was performed using the Tyramide Signal Amplification System (NEN Life sciences, Boston, MA, USA). Sections of 4-*μ*m thickness were deparaffinized and the antigen was retrieved by microwaving in 10 mmol l^−1^ citrate buffer (ph 6.0) for 25 min followed by blocking steps according to the manufacturer's protocol and overnight incubation at 4°C with 1 : 100 dilution of mouse monoclonal antibody 610958 (BD Biosciences, Oxford, UK). After application of the secondary antibody, biotinylated rabbit anti-mouse (DakoCytomation, Ely, UK), the antigen was visualised using diaminobenzidine (DakoCytomation) and the sections were counterstained with haematoxylin, dehydrated and mounted. Substitution of the primary antibody with the identical concentration of mouse immunoglobulin IgG1 (DakoCytomation) served as a negative control. The scoring system was as follows: 0, no nuclear staining; 1, <10% nuclear staining; 2, 10–29% nuclear staining and 3, >30% nuclear staining. The scoring system has been validated and used in other studies of HNSCC (Silva *et al*, 2008).

### Cell lines

Six human HNSCC cell lines (PE/CA-PJ15, PE/CA-PJ41, bhy, hn, jh012, jh019) and HeLa were subject to low ambient oxygen concentrations as follows. Cells were cultured in 5% CO_2_, 20% O_2,_ 75% N_2_ at 37°C in RPMI-1640 supplemented with newborn calf serum (10%) and L-glutamine (1%) and sub-cultured using trypsin-EDTA. For each cell line, three 75-cm^2^ flasks were cultured until 50% confluence was achieved. The first flask of each cell line constituted the experimental control. The second flask was subject to anoxia (0% O_2_, 10% CO_2_, 10% H_2_, 80% N_2_) for 6 h and the third was cultured under anoxic conditions for 24 h. Immediately after completion of the timed incubations, the cells were harvested and DNA and RNA were extracted simultaneously (Allprep DNA/RNA Mini Kit (Qiagen Ltd)). *CYGB* methylation data and *CYGB* and *HIF1A* mRNA expression data were derived as above. Cell lines were cultured on three separate occasions to undertake the experiments in triplicate.

## Results

The range of pathological staging in this series of 37 tumours seemed to be typical of our practice. 35% (13) had cervical lymph node metastases, 22% (8) with extracapsular involvement, 24% (9) had frank mandibular invasion by tumour and 27% (10) had a tumour thickness greater than 20 mm. These features of biological aggression were, as would be expected, interdependent, representing a subgroup of more aggressive tumours.

The MtI, a semi-quantitative measure of methylation for the *CYGB* promoter in the tumour samples varied between 0.00 and 0.54 (mean 0.19). In total, 10 of the tumours had MtI<0.03 and 11 had MtI>0.25. This distribution was consistent with our previous report (Shaw *et al*, 2006) on a larger series of tumour and normal samples. The finding in several other methylated genes that a minimum threshold MtI of approximately 0.25 (i.e. 25% copies of the gene methylated) was required to significantly downregulate expression has been previously noted by our group (Shaw *et al*, 2008) and thus will be applied as a threshold in this analysis

The range of *CYGB* expression for tumour-free ‘normal’ epithelium was narrow (0.48–1.61). When normalised to the mean from this ‘normal’ epithelium, a wide range of *CYGB* mRNA expression was observed in tumours (0.14–10.7; mean 1.38). This represents a 77-fold range in the expression of this gene in the tumours. The data were considered in tertiles, with an underexpressing group (⩽0.5 × normal, *n*=14), and an overexpressing group (⩾1.5 × normal, *n*=11) being individually considered. The control cell line with extensive *CYGB* promoter methylation (CRL 5935, MtI 1.00) had very low levels of *CYGB* mRNA expression (0.02) and the cell line without *CYGB* promoter methylation (HNBE, MtI 0.00) showed relatively high levels of expression (2.96). There was also a wide range of *HIF1A* mRNA expression in the tumours (0.16–12.1; fold range 76; mean 1.70) compared with ‘normal’ epithelium. The range for the normal epithelium was again narrower (0.52–1.53).

### Parameters affecting *CYGB* expression

**Figure 1:**
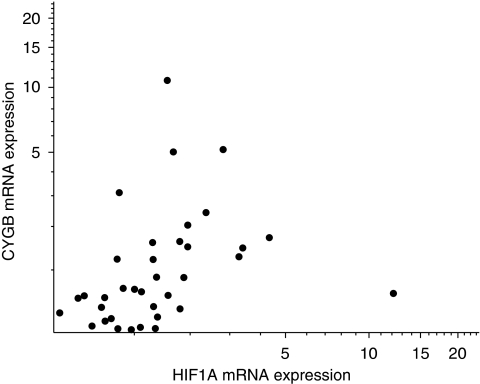
Correlation between *CYGB* mRNA expression and *HIF1A* mRNA expression in head and neck squamous cell carcinoma (HNSCC) tissue samples. (Spearman's rho 0.558, *P*<0.01). Units on the *x* and *y* axes are fold change normalised to the mean of seven normal margin tissue samples (see Materials and Methods).

All those samples with expression of *CYGB* mRNA ⩾1.5 × normal had *CYGB* promoter MtI of <0.25 (Table 1). The data also show that promoter methylation ⩾0.25 was always associated with the downregulation of CYGB. However, other cases had downregulation apparently independent of promoter methylation. A clear positive correlation between mRNA expression of *CYGB* and *HIF1A* mRNA is shown in Figure 1 (*P*<0.01). As may be expected, there was no correlation between *HIF1A* expression and *CYGB* MtI (Spearman's rho −0.10, *P*=0.58). However, those tumours with relatively lower levels of *CYGB* promoter methylation (MtI⩾0.25) showed stronger correlation between expression of *CYGB* and *HIF1A* expression (Table 1).

Borderline significant correlations were observed between *CYGB* expression and clinicopathological features of biological aggression. Overexpression of *CYGB* (⩾1.5 × normal) was associated with extracapsular tumour spread in cervical lymph nodes (63% (5 out of 8) *vs* 21% (6 out of 28), *P*=0.04, Fisher's exact), greater depth of invasion ⩾20 mm (46% (6 out of 13) *vs* 22% (5 out of 23), *P*=0.15, Fishers exact), the presence of cervical lymph node metastases (46% (6 out of 13) *vs* 22% (5 out of 23), *P*=0.15, Fisher's exact), the presence of mandibular invasion by tumour (56% (5 out of 9) *vs* 22% (6 out of 27), *P*=0.10, Fisher's exact) and an Anneroth score ⩾19 (47% (7 out of 15) *vs* 13% (2 out of 15), *P*=0.10, Fisher's exact). No significant correlation with survival or recurrence was found, although there were only five such events in this series of 37 patients and the effect of prescription bias (e.g. confounding survival benefit of post-operative chemoradiation in the most aggressive tumours) must be considered. There was one markedly aggressive tumour (20-mm depth of invasion and 11 positive lymph nodes with extracapsular spread) with notably reduced *CYGB* expression (⩽0.5 × normal) This tumour showed allelic imbalance at two markers close to *CYGB* on 17q25 (data not shown), thus offering a possible explanation for the reduced *CYGB* expression independent of promoter methylation.

HIF1A immunohistochemistry and *HIF1A* mRNA expression for the 32 individual tumours examined were positively correlated, although the significance was borderline (Spearman's rho 0.325, *P*=0.07). The samples with the highest HIFA1 staining (*n*=6; all >30%) had significantly greater *HIFA1* mRNA expression (mean 4.48) compared with the less hypoxic samples (*n*=26; mean 1.04) (Kruskall–Wallis, *P*=0.001). In the samples with *CYG*B MtI<0.25, the correlation between *CYGB* mRNA expression and HIFA1 immunohistochemistry scoring was near significant (Kruskall–Wallis, *P*=0.07), but there was no appreciable trend in the cases with higher values of *CYGB* methylation.

### Human SCCHN cell lines and HeLa

The hn cell line had no detectable *CYGB* promoter methylation (MtI 0.0), bhy and HeLa had intermediate levels of methylation (MtI 0.75 and 0.67) and the remaining cell lines had near 100% methylation (MtI 0.96–0.98). Methylation status was not affected by either 6 or 24 h of anoxic conditions in any of the cell lines (data not shown).

**Figure 2:**
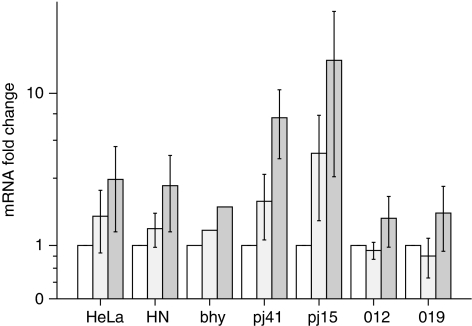
Upregulation of *HIF1A* mRNA with hypoxia for six head and neck squamous cell carcinoma (HNSCC) cell lines and HeLa. Expression is shown relative to expression for each line in 21% O_2_. Unfilled bar, normoxia; pale bar, 6 h of hypoxia; dark bar, 24-h hypoxia. Bar charts indicate mean of five samples with error bars±1 s.e.

**Figure 3:**
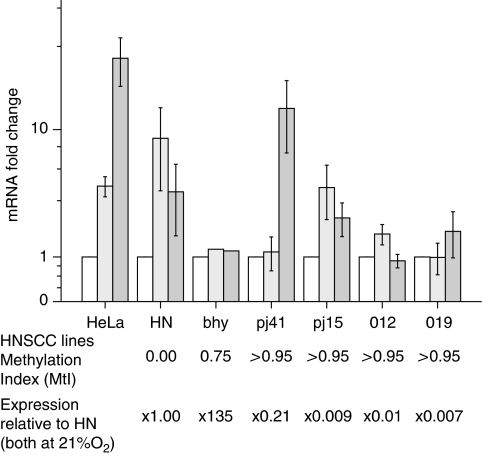
Upregulation of *CYGB* mRNA with hypoxia for six head and neck squamous cell carcinoma (HNSCC) cell lines and HeLa. Corresponding methylation data and expression data relative to the unmethylated HNSCC cell line, hn, are shown below the figure. Expression is shown relative to expression for each line in 21% O_2_. Unfilled bar, normoxia; pale bar, 6 h of hypoxia; dark bar, 24 h hypoxia. Bar charts indicate mean of five samples with error bars±1 s.e.

All the seven cell lines showed an increase in *HIF1A* expression (Figure 2) and a corresponding increase in *CYGB* expression (Figure 3) with longer incubation under anoxic conditions. It should be noted that the absolute levels of *CYGB* mRNA expression (relative to hn) for the cell lines 012, 019 and PE/CA-PJ15 were rather low, as were any incremental increases caused by hypoxia, and that all these cell lines had MtI>0.95. Consequently, the relevance of such minute fluctuations in actual expression might be questioned for these three cell lines. bhy, however, had higher basal levels of *CYGB* expression despite an MtI>0.75 and failed to increase *CYGB* expression in response to hypoxia. Thus, the trend was for increased *CYGB* expression with progressive hypoxia, although the effect was reduced by high baseline *CYGB* expression.

Treatment of the hn, bhy and PE/CE-PJ15 cell lines (which showed differing levels of *CYGB* promoter methylation and baseline *CYGB* expression) with 5-aza-2-deoxycitidine to prevent DNA methylation during DNA synthesis caused upregulation of *CYGB* expression. The degree of upregulation seemed to be dependent on the original level of promoter methylation and/or the original level of *CYGB* expression (PE/CA-PJ41 upregulated >350-fold, bhy upregulated two-fold; data not shown).

## Discussion

In the current study, we have measured *CYGB* gene expression and promoter methylation in a series of head and neck tumours and determined associations with histopathology, clinical characteristics and established markers of tumour hypoxia such as *HIF1A* expression and tumour thickness. We also investigated the *CYGB* and *HIF1A* response of human oral cancer cell lines to hypoxic conditions.

These experiments were performed on a modest series of tumours. Although a multivariate analysis may seem attractive (given the multifactorial hypothesis in question), this analysis would prove underpowered with the data available. It was noted that *CYGB* promoter methylation in the tumour samples never rose above an MtI of 0.54, whereas the majority of the cell lines (five out of six) showed much higher levels of methylation. Correspondingly, high levels of methylation might be detected in micro-dissected tumour tissue compared with our non-microdissected specimens, as it is possible that different cell populations in the tumour show different promoter methylation profiles, thus advocating the use of laser capture microdissection and the analysis of several different cell populations. Similarly, the range of *CYGB* mRNA expression data from individual cell populations might prove even greater that the current data show. There are, however, some technical disadvantages to micro-dissection. The total quantity of DNA obtained is much smaller and PMAs demand relatively high quantity and quality of DNA, particularly as the bisulphite treatment essential to the assay causes a degree of fragmentation.

What remains uncertain is the exact role of the Cygb protein in normal tissue, its level of expression in normal epithelium and underlying stroma, and how this differs in malignancy. Our work in this area has to date been hampered by the apparent lack of specificity of currently available antibodies to the human Cygb protein and its low level of basal expression in normal epithelial cells (data not shown).

Our initial work on promoter methylation found *CYGB* to be significantly methylated in a tumour-specific pattern in around 65% of HNSCC (Shaw *et al*, 2006). This finding, and similar findings in other aerodigestive tract cancers (Xinarianos *et al*, 2006) led us to believe that it has a role as a tumour suppressor gene in HNSCC, perhaps by inhibiting free-radical or stress-induced epithelial hyperplasia. However, the findings we describe in this paper relating to tumour hypoxia and tumour behaviour may seem to suggest that, in established malignancy, *CYGB* takes on a role in relation to the response to tumour hypoxia. Notably, we have observed a significant correlation between extracapsular spread and overexpression of *CYGB* and consistent trends with other prognostic markers, including depth of invasion and lymph node involvement. However, no significant correlation with disease-specific survival or recurrence was found, possibly due to the small number of such events in this series of 37 patients and the confounding survival benefit of post-operative chemoradiation given to the most aggressive tumours.

Our observations lead us to conclude that both hypoxia and promoter methylation influence *CYGB* expression. In the tissue samples, the influence of tumour hypoxia seems greater when *CYGB* promoter methylation is <50% of the maximal levels (i.e. at an MtI of <0.25). However, all 12 of the tumours with *CYGB* MtI⩾0.25 had relatively low levels of *CYGB* mRNA expression and this was irrespective of significant variations in *HIF1A* expression. Thus, assuming some heterogeneity of cell type in the tissue samples examined, promoter methylation seems to have a dominant effect on *CYGB* expression at values of MtI that may represent complete or bi-allelic methylation of the gene (MtI⩾0.25 in this series). The HIFA1 immunohistochemistry data reinforce the correlation seen at the mRNA level. This is significant because regulation of HIF1A occurs by inhibition of degradation of the subunit, as well as at the transcriptional level.

It may be proposed that mutation of the *CYGB* gene is of importance in those tumours that show reduced expression of the transcript but little or no promoter methylation. We have not undertaken mutation screening of *CYGB* in the tumour samples in this study, but we have previously sought, and failed to find, *CYGB* mutations in sporadic oesophageal cancer specimens (McRonald *et al*, 2006). Alternatively, other mechanisms of control could also be proposed, such as histone deacetylation.

Interestingly, the levels of *CYGB* promoter methylation in all seven of the cell lines investigated in the current study were unchanged under culture conditions of reduced oxygen. Unexpectedly, the PE/CA-PJ41 cell line showed an increase (approximately 10-fold) in *CYGB* expression under hypoxic conditions despite an MtI >0.95. However, growth of PE/CA-PJ41 in the presence of a DNA demethylating agent caused *CYGB* expression to increase more than 350-fold, suggesting at least some role for promoter methylation in this cell line. A trend of co-expression of *CYGB* and *HIF1A* was observed in five of the seven cell lines studied under hypoxic conditions. However, the absolute levels of expression of *CYGB* were very low in three of the cell lines, which had promoter methylation >0.95. These data imply that there may be alternate control mechanisms for the activation of this gene and may be explained by observations of hypoxia responsive mRNA stabilisation sites in the gene (Fordel *et al*, 2004a). These trends largely replicate *in vitro* our *ex vivo* cross-sectional tissue data and lend further support to the role of tissue hypoxia in the regulation of *CYGB*.

Hypoxia is a known endogenous marker of poor prognosis in HNSCC and seems to be related to resistance to radiotherapy (Koukourakis *et al*, 2006) and chemotherapy. This is reinforced in our study in which correlations between hypoxia and biological aggression of the tumours (such as depth of invasion, mandibular invasion, involved cervical lymph nodes and extra-capsular spread) were seen. The cellular response to hypoxia seems to be mediated by *HIF1A* in malignancy (Maxwell, 2005) and has been extensively studied in HNSCC (Brennan *et al*, 2005; Quintero *et al*, 2006). Should Cygb prove to be a critical component in the hypoxia response, its manipulation might have therapeutic value, particularly in response to radiation treatment. Is the overexpression of *CYGB* necessary to mediate the hypoxia response, to mediate *VEGF* expression (Fordel *et al*, 2004a), to subtend a survival advantage in more aggressive tumours? Clearly more studies are necessary to clarify exactly where *CYGB* fits in to the cellular hypoxia story and whether it is up- or downstream of *HIF1A*, currently viewed as the orchestrator of the tumour's response to hypoxia. The significance of such studies may not be only in the field of basic science, as the modulation of *HIF1A* is currently a focus for the development of novel radiosensitising drugs. The ongoing controversy of the role of *CYGB* has been recently highlighted by recent suggestions of its function as a tumour suppressor gene in NSCLC (Shivapurkar *et al*, 2008) and HNSCC (Shaw *et al*, 2006), and as a prospective gene therapy (Lv *et al*, 2008) in the prevention of hepatocellular carcinoma after liver cirrhosis.

In this work, we have shown the regulation of *CYGB* expression in HNSCC by both promoter methylation and tumour hypoxia. We have observed that clincopathological measures of a tumour's biological aggression are associated with overexpression of *CYGB* and, in parallel, the overexpression of *HIF1A*. However, we have also observed that, not only does substantial promoter methylation have an overriding effect of *CYGB* downregulation irrespective of hypoxia, but that *CYGB* upregulation in cell lines in response to hypoxia may be less dependent on alterations in *CYGB* promoter methylation status.

### Statistical methods

The data were analysed using SPSS v15.0 software (SPSS Inc., Chicago, IL, USA).

## Figures and Tables

**Table 1:** Interrelationship between *CYGB* methylation, *CYGB* expression and *HIFA* expression in HNSCC tissue samples

		***HIF1A* expression^a^**			
***CYGB* MtI**	***CYGB* expression^a^**	**<1**	**1.0–1.6**	**>1.6**	
<0.25	⩽0.5	4	1	0	Spearman *r*=0.62, *P*=0.002
	0.6–1.4	2	3	2	
	⩾1.5	1	2	8	
⩾0.25	⩽0.5	5	2	1	Spearman *r*=0.32, *P*=0.32
	0.6–1.4	1	2	1	
	⩾1.5	0	0	0	

HNSCC=head and neck squamous cell carcinoma; MtI=methylation index.

a Normalised to the mean of expression in seven normal surgical margin samples (see Materials and Methods).
